# Angong Niuhuang Wan reduces hemorrhagic transformation and mortality in ischemic stroke rats with delayed thrombolysis: involvement of peroxynitrite-mediated MMP-9 activation

**DOI:** 10.1186/s13020-022-00595-7

**Published:** 2022-04-27

**Authors:** Hansen Chen, Yunxia Luo, Bun Tsoi, Bing Gu, Suhua Qi, Jiangang Shen

**Affiliations:** 1 School of Chinese Medicine, The University of Hong Kong, 10 Sassoon Road, Pokfulam, Hong Kong, SAR People’s Republic of China; 2 State Key Laboratory of Pharmaceutical Biotechnology, The University of Hong Kong, Hong Kong, China; 3 School of Medical Technology, Xuzhou Medical University, Xuzhou, 221002 China

**Keywords:** Stroke, Hemorrhagic transformation (HT), Tissue plasminogen activator (t-PA), Angong Niuhuang Wan (AGNHW), Chinese medicine

## Abstract

**Background:**

Hemorrhagic transformation (HT) is a common complication of delayed tissue plasminogen activator (t-PA) treatment for ischemic stroke. Peroxynitrite plays an important role in the breakdown of blood–brain barrier (BBB) and the development of HT. We tested the hypothesis that Angong Niuhuang Wan (AGNHW), a traditional Chinese medicinal formula, could be used in conjunction with t-PA to protect the BBB, minimize HT, and improve neurological function by suppressing peroxynitrite-mediated matrix metalloproteinase-9 (MMP-9) activation.

**Methods:**

We first performed quality control study and chemical identification of AGNHW by using UPLC. In animal experiments, male Sprague–Dawley rats were subjected to 5 h of middle cerebral artery occlusion (MCAO) followed by 19 h of reperfusion plus t-PA infusion (10 mg/kg) at 5 h of cerebral ischemia. AGNHW (257 mg/kg) was given orally at 2 h after MCAO. Hemorrhagic transformation was measured using hemorrhagic scores and hemoglobin levels in ischemic brains. Evans blue leakage was utilized to assess the severity of the blood–brain barrier (BBB) damage. The modified neurologic severity score (mNSS) test was used to assess neurological functions. Peroxynitrite and superoxide was detected by using fluorescent probes. MMP-9 activity and expression were examined by gelatin zymography and immunostaining. The antioxidant effects were also studied by using brain microvascular endothelial b.End3 cells exposed to 5 h of oxygen and glucose deprivation (OGD) plus 5 h of reoxygenation with t-PA treatment (20 µg/ml).

**Results:**

AGNHW significantly reduced the BBB damage, brain edema, reduced hemorrhagic transformation, enhanced neurological function, and reduced mortality rate in the ischemic stroke rats with t-PA treatment. AGNHW reduced peroxynitrite and superoxide in vivo and in vitro and six active chemical compounds were identified from AGNHW with peroxynitrite scavenging activity. Furthermore, AGNHW inhibited MMP-9 activity, and preserved tight junction protein claudin-5 and collagen IV in the ischemic brains.

**Conclusion:**

AGNHW could be a potential adjuvant therapy with t-PA to protect the BBB integrity, reduce HT, and improve therapeutic outcome in ischemic stroke treatment via inhibiting peroxynitrite-mediated MMP-9 activation.

**Graphical Abstract:**

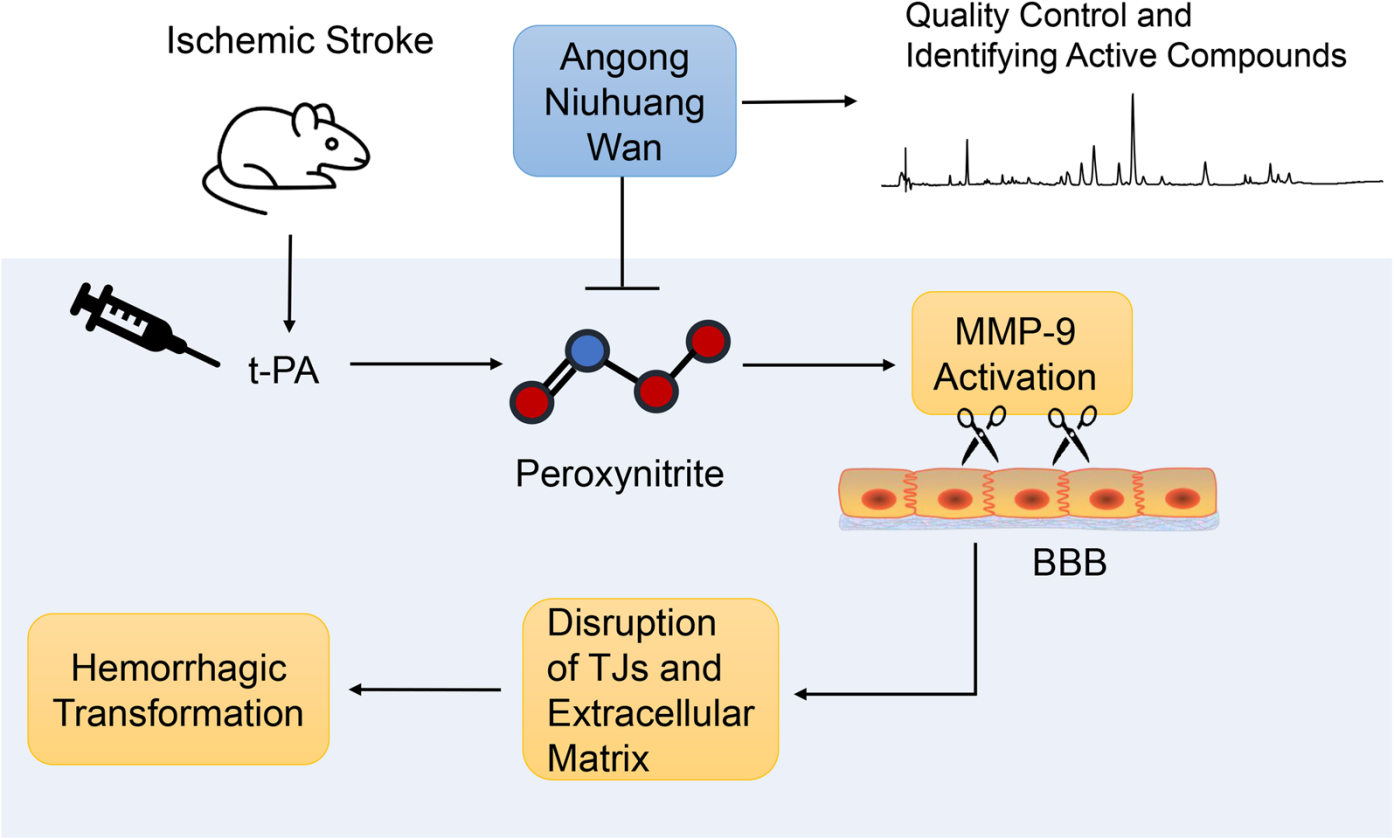

**Supplementary Information:**

The online version contains supplementary material available at 10.1186/s13020-022-00595-7.

## Introduction

Tissue plasminogen activator (t-PA) remains the only thrombolytic drug approved by the U.S. Food and Drug Administration (FDA) for treating acute ischemic stroke, a major disease burden worldwide. However, because of the limited time window of 4.5 h and the complication of hemorrhagic transformation (HT), less than 10% of ischemic stroke patients benefit from t-PA treatment [1–3]. It is critical to develop novel strategies for minimizing HT and extending the therapeutic window of t-PA.

Disruption of the Blood–brain barrier (BBB) is a critical pathological process in t-PA-mediated HT in ischemic stroke [1, 4]. Clinical studies and experimental animal models both support the notion that BBB injury at the early stage of stroke is susceptible to HT after thrombolysis [4–6]. The safe time window for t-PA treatment may differ depending on the severity of the BBB damages [7]. Even if they received t-PA treatment within the 4.5-h time window, some ischemic stroke patients with early BBB disruption could develop HT [7–9]. Consistently, a large multicenter clinical study suggests that accurate measurement of early BBB damage could predict HT in ischemic stroke patients after t-PA treatment [10]. As a result, preserving the BBB is an important strategy for thrombolytic treatment in patients with ischemic stroke [11].

Oxidative stress plays important roles in the BBB disruption and HT in ischemic stroke with delayed t-PA treatment [12, 13]. Recanalization with delayed t-PA treatment generates numerous reactive oxygen species (ROS) and reactive nitrogen species (RNS), medicating cerebral ischemia–reperfusion injury [14]. Peroxynitrite (ONOO^−^) is a representative RNS formed by the instant reaction between nitric oxide (NO) and superoxide (O_2_^.−^). ONOO^−^ has a 400-fold higher than O_2_^.−^, causing protein nitration, lipid peroxidation, and DNA damage [15–18]. Our previous research has shown that ONOO^−^ plays crucial roles in mediating neuronal cell death, BBB damage and HT in ischemic stroke treated with delayed t-PA [13, 19]. Scavenging peroxynitrite inhibited the expression and activities of matrix metalloproteinase (MMPs), protected BBB integrity, minimized HT and improved neurological outcomes in experimental ischemic stroke animal models with or without t-PA treatment [13, 19]. Nitrotyrosine (NT) is a footprint biomarker for ONOO^−^. Thrombolytic therapy increased the levels of NT and MMP-9 in the plasma of ischemic stroke patients [20]. Our recent study indicates that the plasma NT levels are positively correlated with neurological deficit in ischemic stroke patients [21]. Targeting ONOO^−^-mediated MMPs activation is thus a potential strategy for reducing BBB damage and attenuating HT in ischemic stroke with delayed t-PA treatment.

Angong Niuhuang Wan (AGNHW) is a classical medicinal formula used for clinical situations like a stroke in Traditional Chinese Medicine (TCM). AGNHW has been listed in the Chinese Pharmacopoeia for decades. Its main components include Powerdered Buffalo Horn Extract, Bovis Calculus Sativus, Cinnabaris, Coptis chinensis Franch., Artificial Moschus, *Hyriopsis cumingii* (Lea), *Scutellaria baicalensis* Georgi, Realgar, *Curcuma aromatica* Salisb., *Gardenia jasminoides* J.Ellis, and Borneolum Syntheticum (Editorial Committee of Pharmacopoeia of Ministry of Health PR China, 2015). AGNHW was first documented in the Qing Dynasty (seventeenth century) [22], and it has been approved for acute stroke treatment by the China’s State Food and Drug Administration. AGNHW has neuroprotective effects in ischemic stroke patients [23]. Previous studies indicate that AGNHW can reduce infarct volume, attenuate brain edema, and improve neurological functions in animal models of ischemic stroke [24–27]. Our recent studies suggest that AGNHW can scavenge ONOO^−^ and inhibit ONOO^−^-mediated MMPs activation, thereby protecting the BBB integrity, reducing infarct size, and improving the neurological functions in ischemic stroke models [28]. Interestingly, the mineral elements such as realgar and cinnabar are required components to synergistically protect against ischemic brain injury with other herbal materials [29]. Oral administration of AGNHW with regular doses for one week is safe and effective for ischemic stroke treatment [28, 29]. Our systematic review adds to the clinical evidence that AGNHW is safe for acute brain disorders [30]. In the present study, we hypothesized that AGNHW could protect BBB integrity and reduce HT by inhibiting the peroxynitrite-mediated MMP-9 signaling cascades in ischemic brain injury with delayed t-PA treatment. Our findings suggest that AGNHW could be used in conjunction with t-PA treatment to reduce HT and improve stroke outcomes.

## Materials and methods

### Quality control of AGNHW

#### Chemicals and materials

AGNHW was obtained from Beijing Tong Ren Tang Co., Ltd., (Z11020959, 3 g/pill). The Millipore Milli Q-Plus system was used to prepare the deionized water (Merk, Mini Q ZQ7000). VWR supplied the ethanol, and DUKSAN Ltd supplied the acetonitrile (HPLC gradient). Geniposide, wogonoside, wogonin and berberine with the purity of 98% were purchased from Chengdu Ruifensi Biotechnology Co., Ltd.; Baicalein, palmatine hydrochloride, epiberberine and coptisine with the purity of 98% were obtained from Nanjing Yuanzhi Biotechnology Co., Ltd.; Baicalin with the purity of 95% was purchased from Sigma. Additional file 1 lists the chemical structures of these reference compounds.

#### AGNHW extraction

We compared various extraction methods for AGNHW using various solvents. The entire AGNHW pill was placed in the grinding crucible and homogeneously pulverized using liquid nitrogen. AGNHW (10 mg) was accurately weighed and ultrasonically extracted for 1 h at power 250 W and frequency 45 kHz with 1 ml solvent (distill water, or 30% ethanol, or 50% ethanol, or 70% ethanol, or 100% ethanol). After cooling in a water bath for 5 min, the samples were centrifuged at 10,000 rpm and the supernatant was filtered (0.2 µm). For HPLC analysis, a 100 μL aliquot of was diluted with 900 μL of 70% Ethanol. The final concentration was 1 mg/ml of AGNHW. We chose the 70% ethanol extract for quality control of AGNHW and in vitro study based on the extraction efficiency results.

#### Quality control analysis with ultra high-performance liquid chromatography (UPLC)

Quality control for AGNHW was performed by using ultra-high performance liquid chromatography (Thermo-Scientific, Ultimate-3000) system, controlled by Thermo Scientific Chromeleon 7.2 software. The separation of 9 analytes was accomplished using an ACE-C 18 HPLC column (4. 6 mm × 250 mm, 5 μm) at temperature 30 °C. As shown in Additional file 2, the gradient elution is optimized. The injection volume is 10 µl and the nine analytes were monitored simultaneously at 240 nm. We first validated the method's precision, reproducibility, accuracy, stability, and linearity before quantitatively analyzing the AGNHW extract. Six replicate injections of samples were used to assess precision. Six independently prepared samples were analyzed to determine reproducibility. he stability was determined by analyzing the same sample every 3 h for five times at room temperature. The recovery test was used to determine the method's accuracy by using marker compounds. The average percent of recoveries was calculated by dividing the detected amount by the added amount. To detect linearity, 70% ethanol stock solutions of nine reference compounds were diluted to a series of concentrations, 100, 50, 25, 12.5, 6.25, 3.125, and 1.5625 µg/ml, for the construction of calibration curves. To create the standard curve, each concentration was examined in triplicate. These standard curves were used to analyze a total of 9 compounds in AGNHW samples.

### ONOO^−^ scavenging assay

We tested whether AGNHW extract could react with ONOO^−^ directly by using UPLC analysis. First, we recorded the HPLC chromatograms of AGNHW extract (1 mg/ml). The AGNHW extract solution was then treated with various concentrations of ONOO^−^ (50 µM, 100 µM, 200 M, 400 µM, 800 µM, 1600 µM). We quantitatively identified the compounds reacting with ONOO^−^ by comparing the retention time and UV absorbance with the standard compounds, demonstrating the dose-dependent reduction of peak after adding various concentrations of ONOO^−^ solution.

### Middle cerebral artery occlusion (MCAO) model

Male Sprague–Dawley (SD) rats (290–310 g) were obtained from the Laboratory Animal Unit, The University of Hong Kong. The Committee on the Use of Live Animals in Teaching and Research at The University of Hong Kong approved all animal experimental protocols. Experiments were carried out in accordance with national and institutional ethics and biosafety guidelines. The rats were kept in humidity and temperature-controlled environment with a 12-h light/dark cycle. To mimic a prolonged stroke, rats were subjected to 5 h of middle cerebral artery occlusion (MCAO) followed by 19 h of reperfusion [19]. Following isoflurane anesthesia, the skin of rat’s neck was opened to expose the common carotid artery (CCA), external carotid Silicon (ECA) and internal carotid artery (ICA). From the ECA to the ICA, a silicone coated suture (Doccol, Redlands, CA, USA) was inserted until it occluded the origin of the middle cerebral artery (MCA). The same procedure was performed on sham control rats but without MCA occlusion. The duration of anesthesia was recorded (Additional file 3A). Rats’ body temperature was monitored during the surgery and recovery using an infrared thermometer (Additional file 3B). To allow for reperfusion, the suture was removed 4.5 h after MCAO. To confirm the success of the brain infarct, a thin slice of brain tissue was stained with 2,3,5-triphenyl-2H-tetrazolium chloride (TTC) [31].

### Experimental designs and drug treatment

SD rats were randomly assigned to one of four groups: Sham, MCAO 5 h plus reperfusion 19 h (M5/R19), M5/R19 plus t-PA, M5/R19 plus t-PA and AGNHW treatment. Each group had at least ten rats. To simulate early intervention, AGNHW dissolved in saline (257 mg/kg) was administered orally 2 h after MCAO. By converting the body surface area of humans to rats, this dosage is equivalent to a daily dosage in human subjects (3 g/pill per day) [32]. Saline was used as a vehicle control. The rats were given t-PA (10 mg/kg, Actilyse, Boehringer Ingelheim) or saline via the femoral vein at 4.5 h after MCAO ischemia, with a 10% bolus followed by a 90% slow infusion within 0.5 h, defining the infusion time at 5 h after MCAO. In this study, the outcome was measured by a researcher who was not aware of the grouping or surgical procedure.

### Mortality rates and neurological deficit scores

We calculated the mortality rates 24 h after the onset of an ischemic stroke. The dead rats were not subjected to any further testing. The modified neurologic severity score (mNSS) test was used to assess each group's neurological deficit. The mNSS test comprises the motor, reflex, and balance tests described in our previous publication: the score ranges from 0 to 18, with 0 representing normal and 18 representing maximal neurological deficit [19].

### Brain edema measurement

At 24 h after stroke, rats were deeply anesthetized with isoflurane and subjected to intracardiac perfusion with ice-cold PBS to remove circulating blood. After that, rat brains were collected and cut into 2-mm thick coronal slices. The brain slices were photographed digitally and then analyzed using Image J software (National Institutes of Health). A brain swelling index was calculated by dividing the ischemic side area by the non-ischemic side area [33].

### Evans blue test for BBB leakage

The amount of Evans blue (EB) extravasation into ischemic brain indicates the severity of BBB leakage [34]. Under anesthesia, rats were intravenously injected with 2% EB (3 ml/kg, Sigma); 1 h later, rats were transcardially perfused with 250 ml PBS to completely remove the circulating EB. The brain tissues were then harvested and cut into 2-mm thick coronal sections in a series. EB extravasation in brain tissues was digitalized and tissues were frozen for further study. To determine the amount of EB in brain tissue, we homogenized and sonicated the tissue PBS before adding the same volume of 50% trichloroacetic acid (Sigma). Supernatants were collected after 20 min of centrifugation at 15,000 rpm to determine the OD value at 620 nm using a microplate reader (Bio-Rad, Hercules, CA, USA). The amount of EB was calculated using an EB standard curve, and extravagated EB dye was quantified as g/g brain tissue.

### Hemorrhagic transformation measurement

The severity of hemorrhage was assessed using hemorrhagic scores and quantified using hemoglobin levels, as recommended by experts [35]. Hemoglobin in the brain parenchyma represents the volume of extravasated red blood cells. After transcranial perfusion, each brain hemisphere was collected and homogenized with cold PBS and sonicated on ice for 30 s, followed by a 15-min centrifuge at 15,000*g* to collect the supernatant. The level of hemoglobin in the supernatant was determined using a hemoglobin assay kit (BioAssay Systems, Hayward, CA, USA), yielding an optical density value with a microplate reader at 400 nm (Bio-Rad). Each sample’s hemoglobin concentration was calculated as (OD sample − OD blank)/(OD standard − OD blank) 200 (mg/dL). To assess hemorrhagic scores, we cut rat brains into 2-mm coronal slices and photographed them digitally. We previously described how hemorrhages were macroscopically classified into five severity levels [36].

### Peroxynitrite and superoxide detection in ischemic brain tissue

At 24 h after MCAO, brain tissues were collected and immediately frozen in embedding medium (Leica). To detect peroxynitrite, frozen brain sections (20 µm) were rapidly prepared and incubated with our specific fluorescent probe HKYellow AM [37]. To detect superoxide separately, a commercially available superoxide probe HEt (Thermo Fisher Scientific) was used. At room temperature, either probe was incubated at a concentration of 20 µM for 30 min. The samples were then fixed with 4% PFA, and fluorescent signals from the probes were detected using the Carl Zeiss LSM 880 confocal microscope.

### Cell culture and treatment

To investigate the effect of AGNHW on peroxynitrite scavenging in vitro, we used brain microvascular endothelial b.End3 cells from the American Type Culture Collection (ATCC) and cultured them in a humidified atmosphere with 5% CO_2_ and 95% air at 37 °C. Cell culture medium was high glucose Dulbecco’s modified Eagle’s medium (DMEM, Gibco, USA) with 10% fetal bovine serum (Gibco, USA) and 1% penicillin–streptomycin (Life Technologies).

b.End3 cells were subjected to oxygen and glucose deprivation (OGD) followed by reoxygenation to simulate oxidative stress injury. During OGD treatment, the cell culture medium was replaced with the same medium but without glucose and placed at 37 °C in a humidified airtight chamber saturated with 95% N_2_/5% CO_2_. We wanted the oxygen concentration to be less than 1%, as measured by an oxygen analyzer (Sable Systems, Las Vegas, NV, USA). Cells in the control group were incubated in normal DMEM medium at 37 °C in a humidified incubator with 5% CO_2_ and 95% room air. After 5 h of OGD treatment, cells were reoxygenated and incubated for another 5 h in normal DMEM medium. During reoxygenation, cells were treated with t-PA (20 µg/ml) or the same volume of vehicles. b.End3 cells were also treated with AGNHW extract (50 µg/ml, 100 µg/ml) or vehicle.

### Peroxynitrite and superoxide detection in vitro

We used hydroethidine (HEt) and HKYellow-AM fluorescent probes to detect superoxide and peroxynitrite in b.End3 cells, similar to in vivo detection. The b.End3 cells were incubated with HEt and HKYellow-AM (10 µM) separately at 37 °C for 20 min after 5 h of OGD plus 5 h of reoxygenation, with or without t-PA treatment or AGNHW treatment. A fluorescence microscope (Carl Zeiss) with an Axio Vision digital imaging system was used to detect the fluorescent signals.

### Immunofluorescence

Immunostaining was used to look at the expression of 3-NT, matrix metalloproteinase-9, tight junction protein claudin-5, and extracellular matrix collagen IV in brain tissues 24 h after a stroke. Frozen brain sections were blocked with 5% goat serum (Thermo Fisher Scientific) for 1 h before being incubated with primary antibodies overnight at 4 °C: anti-3-NT (Abcam, 1:50), MMP-9 (Santa Cruz, 1:100), Claudin-5 (Thermo Scientific, 1:200), and Collagen IV (Thermo Fisher Scientific) (Abcam, 1:800). Secondary antibodies were incubated at room temperature for 2 h, including Alexa Fluor 568 Goat anti-mouse (Invitrogen), Alexa Flor 488 Goat anti-rabbit (Invitrogen), Alexa Flor 647 Goat anti-mouse (Invitrogen). The nucleus was stained with DAPI. Fluorescence signals were detected and captured using a Carl Zeiss LSM 780 confocal microscope system.

### Western blot analysis

Protein was extracted from brain hemispheres on ice using RIPA buffer containing 1% protease and a phosphorylate inhibitor cocktail. We loaded the same amount of total protein for electrophoresis after quantifying the protein concentration, and then transferred the protein to polyvinylidene fluoride (PVDF) membranes (Millipore, Billerica, MA, USA). After blocking with 5% bovine serum albumin (BSA) for 1 h, the membranes were incubated overnight at 4 °C with the following primary antibodies: β-actin antibody (Cell signaling, 1:4000), p47 phox (Santa Cruz, 1:200), p67 phox (Santa Cruz, 1:200), iNOS (Abcam, 1:500), 3-NT (Abcam, 1:500). HRP-conjugated secondary antibody (Cell Signaling Technology) was incubated with the membrane at room temperature for 2 h. Protein bands were detected using ECL Advance (GE Healthcare Bio-Sciences, USA) with the Bio-Rad system. Image Lab software was used to calculate the intensities of all bands.

### Gelatin zymography

The MMP-9 band activity on gelatin gels was investigated using gelatin zymography [38]. Brain native protein samples were loaded in 10% acrylamide gel containing 1 mg/ml gelatin (Sigma) for electrophoresis. Following electrophoresis, gels were washed 2.5% Triton-100 to remove SDS before being incubated with developing buffer at 37 °C for 48 h. After Coomassie blue staining, active MMP-9 was visible as a transparent band. Wash the gels with destain buffer (40% methanol, 10% acetic acid, and 50% ddH2O) until the MMP-9 bands became sharp. The MMP-9 was photographed digitally, and the intensity of the bands was analyzed using ImageJ software (NIH, Bethesda, MD, USA).

### Measurement of t-PA activity

We examined whether AGNHW affects the t-PA activity by using a commerical assay kit (Abcam), with the manufacture provided protocol. The kit's standard t-PA can convert plasminogen to plasmin, which can then be quantified to reflect its activity. We tested whether AGNHW extracts affect t-PA activity by adding water extract or ethanol extract to the reaction system at a final concentration of 100 µg/ml. As a control, the same volume of blank solution was used. At 24 h after the reaction, the OD value was measured with a microplate reader at a wavelength of 405 nm (BioRad).

#### Statistical analysis

Using GraphPad Prism, we performed a one-way ANOVA followed by a post-hoc Tukey test on the data. The statistical significance level was set at P 0.05. In graphs, all data were expressed as Mean ± SEM.

## Results

### Quality control and chemical identification in AGNHW extract

**Fig. 1:**
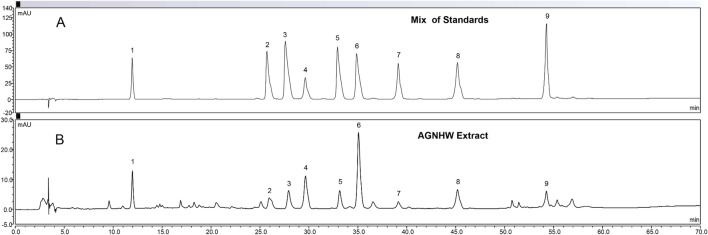
Representative HPLC chromatograms of mixed standards and an extract of Angong Niuhuang Wan (AGNHW). **A** chromatographic profiles of nine mixed standards: Peak 1, Geniposide; Peak 2, Epiberberine; Peak 3, Coptisine; Peak 4, Baicalin; Peak 5, Palmatine; Peak 6, Berberine; Peak 7, Wogonoisde; Peak 8, Baicalein; Peak 9, Wogonin. **B** AGNHW extract HPLC profile. The peaks correspond to those found in mixed standards

We first developed a UPLC method for quality control study which had a good relationship between concentrations and the peak areas of the analytes within the test ranges (R^2^ ≥ 0.999 (Additional file 4). The overall RSDs for the precision and repeatability tests were less than 0.53% and 0.95%, respectively. To prepare the AGNHW extract, we optimized the extraction method by comparing the extracts of AGNHW with ethanol (30%, 50%, 70%, 100%) or 100% dd H_2_O and 70% ethanol yielded the highest extraction efficacies for each compound and the total AUC area (Additional files 5 and 6). Thus, the 70% ethanol exaction was used and the batch-to-batch consistency for three batches was monitored by determining chemical compounds according to retention times and reference chemicals of the UV spectra. The typical chromatograms of the reference compounds (A) and AGNHW samples (B) was depicted in Fig. 1 and the contents of these compounds in the AGNHW samples were shown in Table 1. We identified total nine representative compounds in the AGNHW extract including geniposide, epiberberine, coptisine, baicalin, palmatine, berberine, wogonoside, baicalein and wogonin. The RSDs of the peak areas of those analytes was detected within 24 h were less than 4.93% in the stability test. For all analytes, the accuracy rates with spike recoveries were 96.90–105.19% at low concentration, 95.36–103.30% at medium concentration, and 95.03–105.24% at high concentration. For the AGNHW extract, the overall RSDs of inter-day variations and the repeatability were less than 4.34% and 4.79% respectively (Table 1). In the stability test, the RSDs of the peak areas for AGNHW sample detected within 24 h were lower than 3.90%. Thus, the quality control and chemical identification are reliable,

**Table 1:** Precision, stability and repeatability of the Angong Niuhuang Wan sample, and the contents of nine compounds

Sample	Precision (RSD, n = 6) (%)	Stability (RSD, 24 h) (%)	Repeatability (RSD, n = 6) (%)	Contents in AGNHW
Geniposide	0.93	0.79	3.74	5.799 ± 0.033 mg/pill
Epiberberine	3.77	3.28	4.79	1.844 ± 0.045 mg/pill
Coptisine	1.02	0.97	4.79	1.752 ± 0.167 mg/pill
Baicalin	1.36	1.16	3.88	10.42 ± 0.214 mg/pill
Palmatine	1.22	1.14	3.86	1.856 ± 0.009 mg/pill
Berberine	0.78	0.67	3.75	9.258 ± 0.021 mg/pill
Wogonoside	4.34	3.90	3.86	1.153 ± 0.043 mg/pill
Baicalein	1.66	1.62	3.55	3.454 ± 0.064 mg/pill
Wogonin	2.14	1.97	3.56	1.340 ± 0.036 mg/pill

providing reliable chemical consistency for the studies.

### AGNHW decreased mortality rates and ameliorated neurological deficit in transient cerebral Ischemia rats with delayed t-PA infusion

**Fig. 2:**
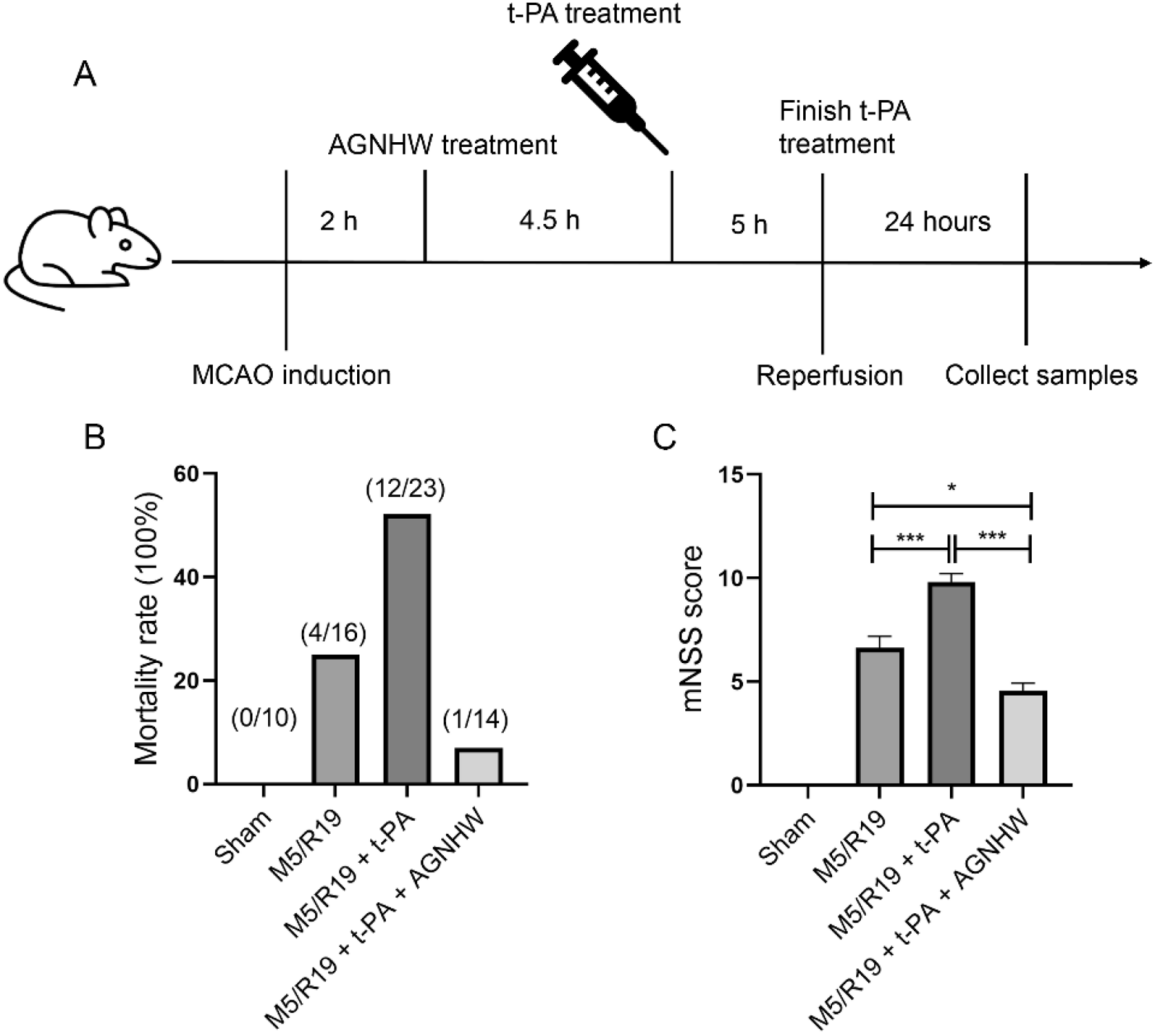
AGNHW treatment decreased mortality rates and improved neurological deficit in transient ischemic stroke treated with delayed t-PA. Sham, sham operation with no MCA ligation; M5/R19, 5 h of MCAO plus 19 h of reperfusion with saline as vehicle treatment (1 ml). M5/R19 + t-PA, rats were treated with t-PA infusion (10 mg/kg) for half an hour started from 4.5 h after MCAO, and followed by 19 h of reperfusion; M5/R19 + t-PA + AGNHW, rats were subjected to M5/R19 + t-PA (10 mg/kg dissolved in 1 ml saline) with the treatment of AGNHW (257 mg/kg) orally at 2 h after MCAO. **A** Diagram summarizing the design of the animal study. **B** Mortality rates: Mortality was calculated at 24 h after MCAO operations. Delayed t-PA treatment increased the mortality rate in the M5/R19 rats which was mitigated by AGNHW treatment. **C** Neurological deficit scores: The mNSS was used to account for neurological deficit scores at 24 h after MCAO operations. The M5/R19 + t-PA rats had increased the mNSS value which was reduced by AGNHW treatment. *P < 0.05, ***P < 0.0001, n = 10–13

We then investigated the effects of the AGNHW extract on the primary and secondary outcomes by counting mortality rates and neurological deficit scores respectively in a transient cerebral ischemic rat model with delayed t-PA treatment. The transient cerebral ischemic rat model was induced by 5 h of MCAO ischemia and followed by 19 h of reperfusion with the t-PA infusion at 5 h after ischemic onset. Sham control rats were received similar surgical operation without ligation of MCAO or t-PA infusion. The dosage of AGNHW extract (257 mg/kg), equivalent to the daily dosage of human subjects with provided safe and efficacy [28, 29], was orally administrated to the M5/R19 + t-PA rats at 2 h after MCAO ischemia (M5/R19 + t-PA + AGNHW). The vehicle treated M5/R19 rats with or without t-PA infusion were labelled as M5/R19 + t-PA and M5/R19 respectively in the figures, which were received normal saline treatment. As shown in Fig. 2, the mortality rates were at 25% in the M5/R19 group but increased to 50% in the M5/R19 + t-PA group. Notably, the mortality rates were reduced to 7% in the M5/R19 + t-PA + AGNHW group. These results suggest that AGNHW could reduce the mortality rates in the transient cerebral ischemia rats with delayed t-PA infusion. We then evaluated the neurological deficit scores (mNSS) in the survived MCAO rats (Fig. 2C). Similarly, the mNSS scores were significantly increased by the delayed t-PA treatment at 5 h in the transient cerebral ischemia rats (M5/R19 + t-PA group). The AGNHW treatment not only abolished the t-PA-induced increase of the mNSS scores but also lead to the mNSS scores significantly lower than the MCAO rats without t-PA treatment. These results suggest that AGNHW could be an effective adjunct therapy with t-PA to reduce mortality and ameliorate neurological dysfunctions in ischemic stroke treatment.

### AGNHW attenuated BBB damage, brain edema, and hemorrhagic transformation (HT) in transient cerebral ischemia rats with delayed t-PA infusion

**Fig. 3:**
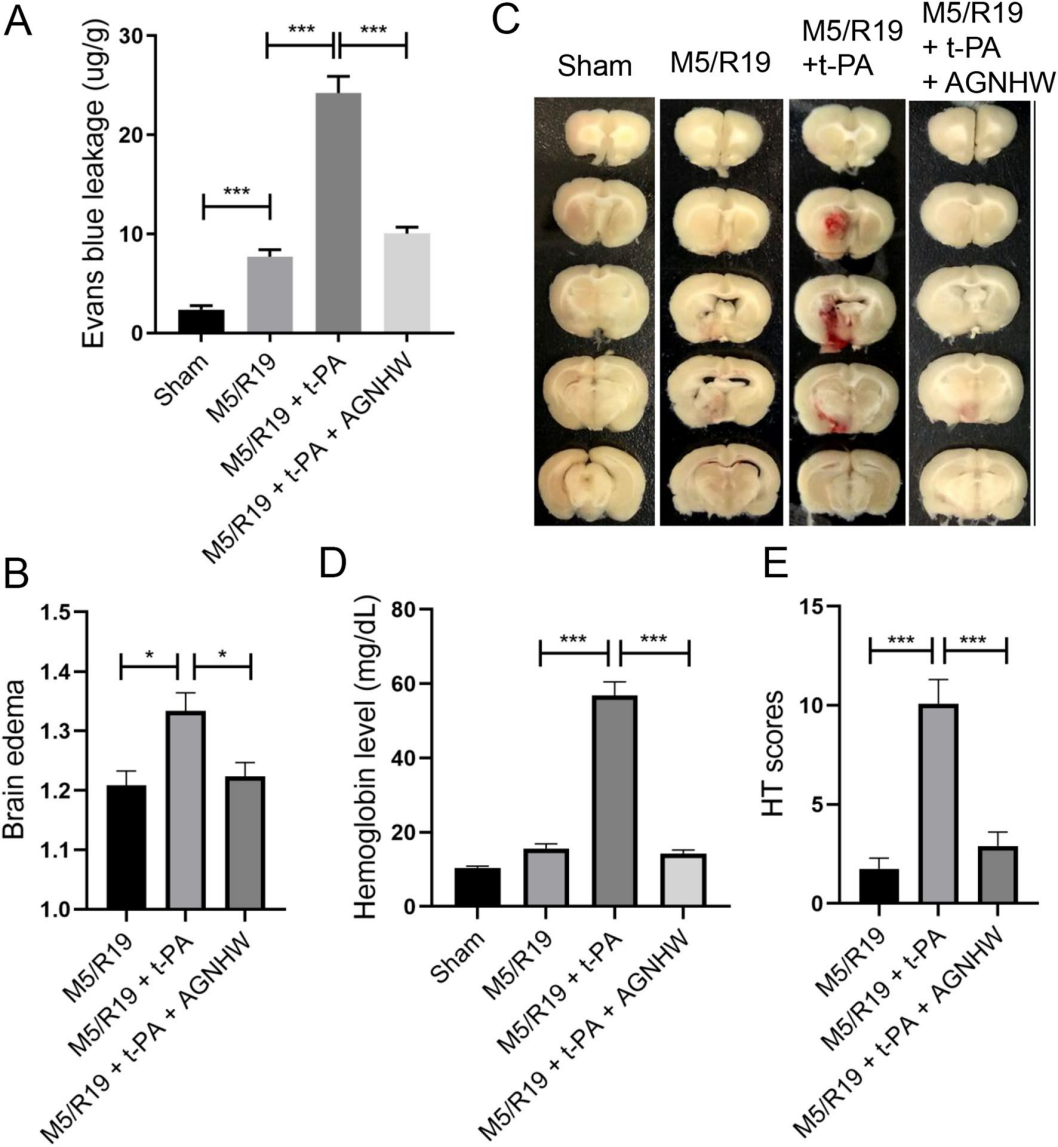
AGNHW treatment preserved the BBB integrity, attenuated brain edema and decreased hemorrhagic transformation (HT) in transient ischemic stroke with delayed t-PA treatment. **A** Evans blue leakage assay was used to assess the severity of BBB injury in the ischemic hemispheres at 24 h after MCAO. n = 5. **B** Quantitative analysis of brain edema: The brain edema was quantified by dividing the total area of ischemic side by the total area of non-ischemic side at 24 h after MCAO. n = 10. **C** Representative HT slices from ischemic brains. **D** Quantitative assessment of hemoglobin levels in the ischemic brains to determine HT severity, n = 6. **E** hemorrhagic scores HT scores as another measure for HT, *P < 0.05, *** P < 0.001, n = 10 per group

We then assessed the effects of AGNHW on the BBB permeability, brain edema, and HT in MCAO ischemic rats with delayed t-PA treatment (Fig. 3). Evans blue leakage assay revealed that the t-PA infusion aggravated the BBB permeability in the M5/R19 group as indicated by the Evans blue extravasation into brain parenchyma. The treatment of AGNHW abolished the t-PA-induced BBB disruption and brain edema in the rats (Fig. 3A, B). Meanwhile, the M5/R19 + t-PA group had the increased incidence and severity of HT in the ischemic brains, showing hemoglobin extravasation into brain tissues and the increased HT scores. The treatment of AGNHW prevented the inductions of the hemoglobin extravasation and decreased hemorrhagic scores in the M5/R19 rats with delayed t-PA treatment (Fig. 3C–E). Furthermore, AGNHW treatment had no effect on the fibrinolytic activity of t-PA (Additional file 7). Thus, AGNHW could protect the BBB integrity, reduce cerebral edema, and minimize HT in the transient cerebral ischemic rats with delayed t-PA treatment.

### AGNHW inhibited ONOO^−^ production in ischemic brains with delayed t-PA treatment and hypoxic brain microvascular endothelial cells (BMECs)

**Fig. 4:**
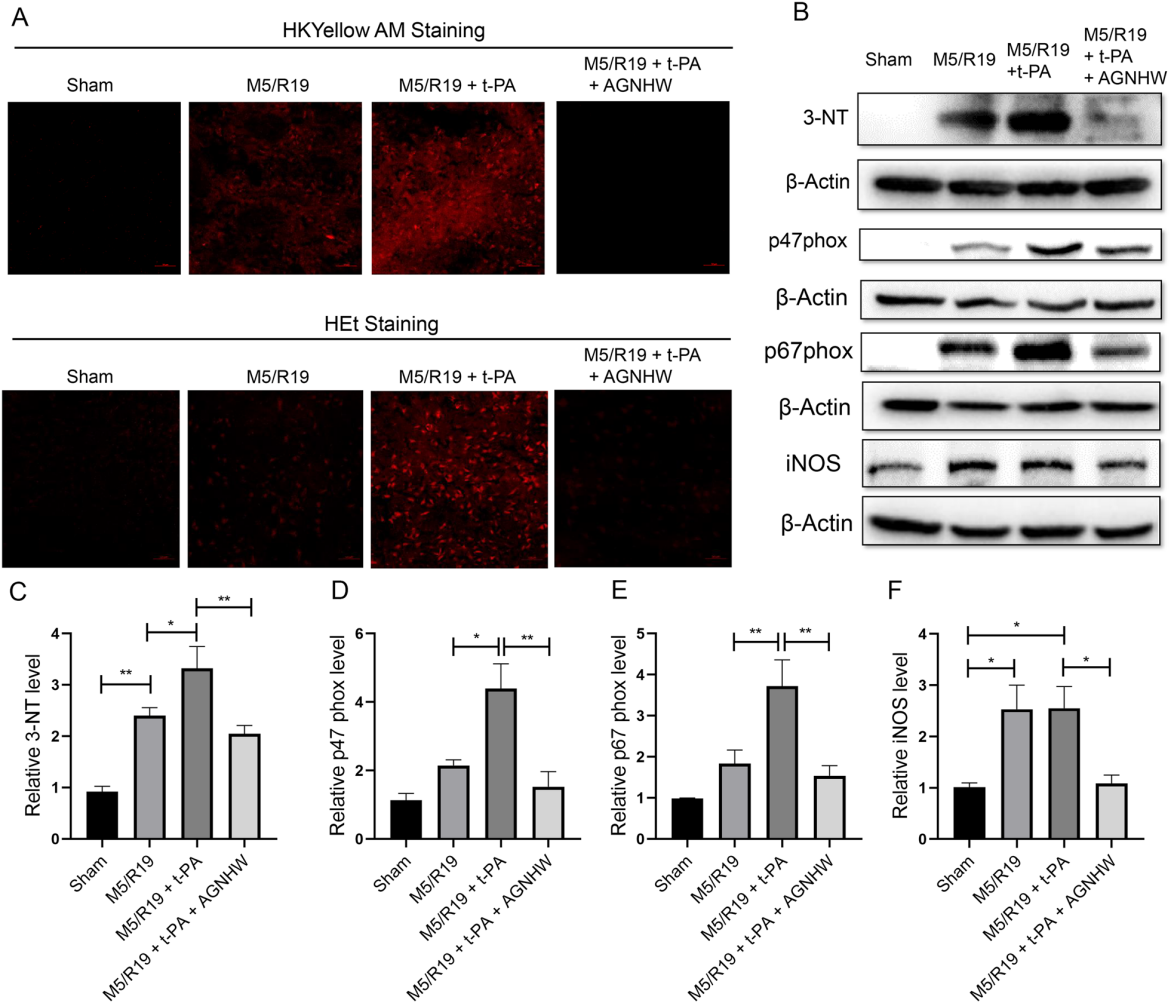
AGNHW treatment reduced the levels of peroxynitrite and superoxide, as well as the expression of NADPH oxidase subunit p47phox and p67 phox, and 3-nitrotyrosine (3-NT) in transient ischemic stroke rat model with delayed t-PA treatment. **A** Peroxynitrite (ONOO^−^) and superoxide (O_2_^.−^) levels in the brain tissues: ONOO^−^ and O_2_^.−^ were detected with HKYellow AM and HEt probe staining respectively in the ischemic brains at about 6 mm away from the frontal tip. **B** Representative western blot results for the expression of 3-nitrotyrosine (3-NT), p47phox, p67phox, and iNOS in the ischemic brain hemispheres. **C** Statistical analysis of 3-NT expression. **D** Statistical analysis of p47phox expression. **E** Statistical analysis of p67phox expression. **F** Statistical analysis of iNOS expression. *P < 0.05, **P < 0.01, n = 10 per group

**Fig. 5:**
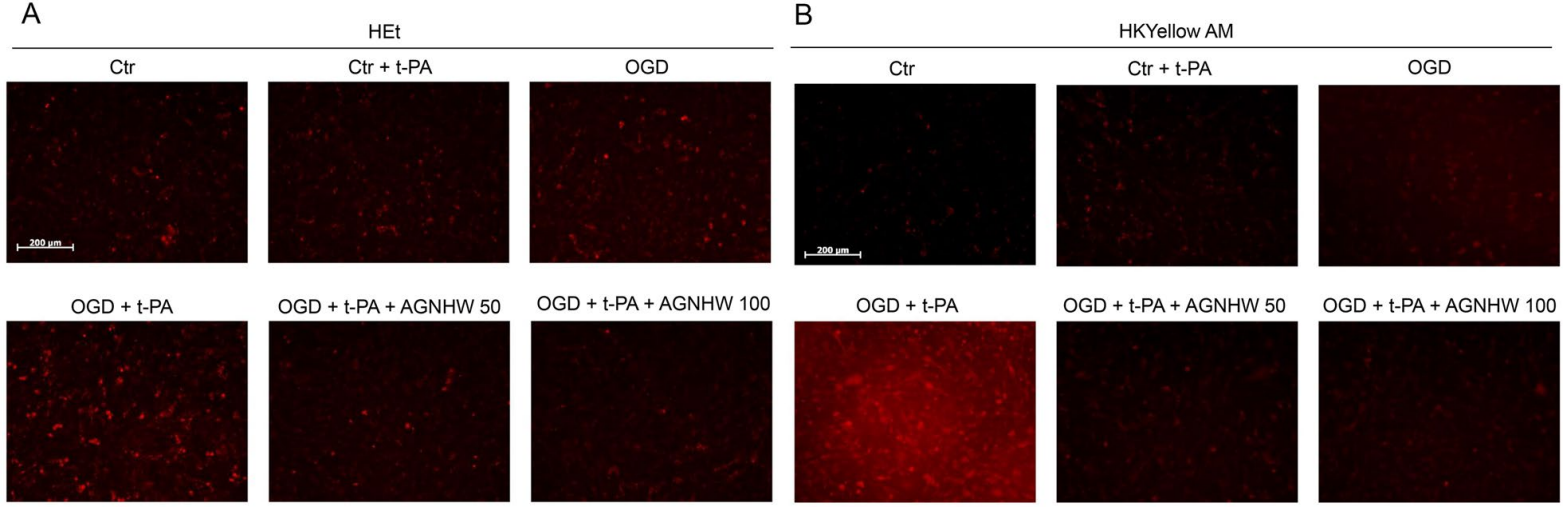
AGNHW treatment inhibited peroxynitrite and superoxide fluorescence in cultured brain microvascular endothelial b.End3 cells in vitro. Ctr, cells were incubated under normoxic condition; OGD, cells were exposed to 5 h of oxygen and glucose deprivation followed by 5 h of reoxygenation; Ctr + t-PA, cells were treated with t-PA (20 µg/ml) under normoxic condition; OGD + t-PA, cells were exposed to 5 h of oxygen and glucose deprivation followed by 5 h of reoxygenation, and the cells were treated with t-PA (20 µg/ml) upon reoxygenation; OGD + t-PA + AGNHW: Cells were subjected to 5 h of oxygen and glucose deprivation 5 h of reoxygenation before being co-treated with t-PA (20 µg/ml) and AGNHW (50, 100 µg/ml) upon reoxygenation. HKYellow AM and HEt staining were used to detect ONOO^−^ and O_2_^.−^, respectively. **A** Representative fluorescent image of HEt staining for O_2_^.−^ in b.End3 cells. **B** Representative fluorescent image of HKYellow AM staining for ONOO^−^ in b.End3 cells in vitro

Our previous studies indicate that ONOO^−^ is a key factor in MMPs activation, BBB degradation and HT in ischemic stroke with the delayed t-PA treatment [13, 19]. Scavenging ONOO^−^ could suppress MMPs activity, protect the BBB integrity, and prevent HT in ischemic stroke rat models with delayed t-PA treatment [12, 13, 19]. To detect ONOO^−^ directly, we used a rhodamine-based fluorescent probe HKYellow AM to detect the production of ONOO^−^ in the ischemic brains. Our previous studies indicate that HKYellow AM has high sensitivity, specialty, and reliability to detecting ONOO^−^ in both cellular and tisuuses [19, 37]. The results revealed a significant increase of ONOO^−^ production in the ischemic brains with delayed t-PA treatment. The ONOO^−^ induced fluorescence was remarkably suppressed by AGNHW treatment (Fig. 4A). We also used hydroethidine (HEt) fluorescent probe to detect O_2_^−^ production. The delayed t-PA treatment elevated O_2_^−^ levels in the ischemic brains, which was remarkably reduced by AGNHW treatment (Fig. 4A). AGNHW extract also down-regulated the expression of 3-NT, further confirming the inhibition of ONOO^−^ production in the ischemic brains (Fig. 4B, C). To explore whether AGNHW extract could inhibit the production of superoxide and nitric oxide, the parent free radicals, we examined the expressions of NADPH oxidase subunits and inducible nitric oxide synthase (iNOS). As expected, AGNHW extract inhibited the expression of NADPH oxidase subunits p47phox and p67phox, and iNOS in the ischemic brains with the delayed t-PA treatment (Fig. 4B, D–F). We then performed the in vitro experiments to further confirm the antioxidant property by using BEMCs, which were exposed to OGD for 5 h followed by reoxygenation for 5 h (OGD/R) and t-PA treatment (20 µg/ml). The HEt and HKYellow AM probes were adopted to detect O_2_^−^ and ONOO^−^ respectively. Consistently, exposures to OGD/R plus t-PA treatment significantly increased the productions of O_2_^−^ and ONOO^−^ in the cells which were abolished by AGNHW extract (50 µg/ml, 100 µg/ml) (Fig. 5A, B; Additional file 8). Those results suggest that AGNHW extract has the bioactivities of scavenging and inhibiting O_2_^−^ and ONOO^−^ in vitro and in vivo.

### AGNHW inhibited MMP-9 expression and activity, and preserved collagen IV and tight junction protein claudin-5 in ischemic brains with delayed t-PA treatment

**Fig. 6:**
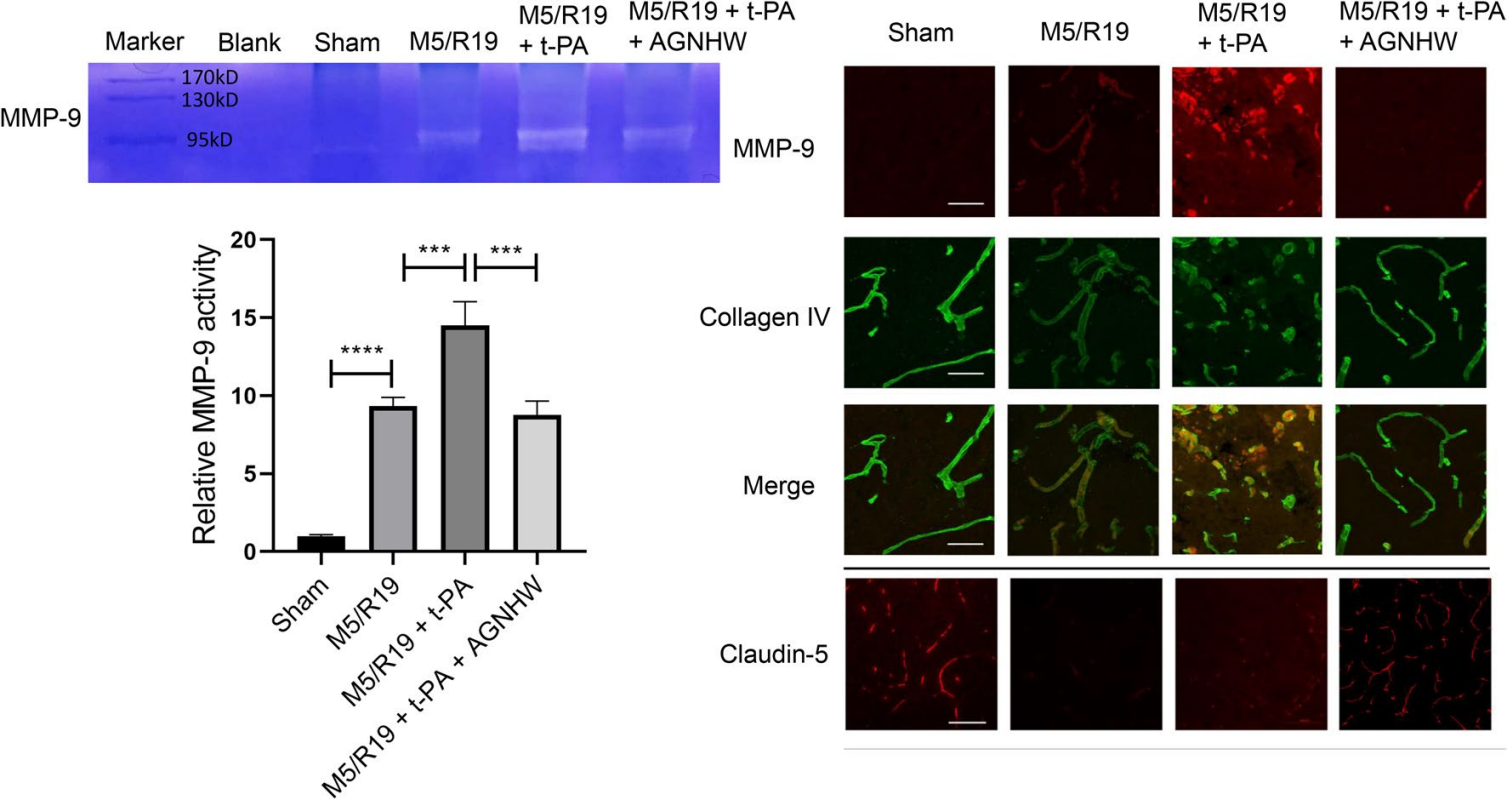
In a transient ischemic stroke rat model with delayed t-PA treatment, AGNHW treatment suppressed MMP-9 expression and activity, maintained microvascular integrity, and reserved the expression of collagen IV and tight junction protein claudin-5. **A** Representative gelatin zymography graph demonstrating MMP-9 activity in protein extracts from ischemic hemispheres. **B** Statistical analysis of MMP-9 activity of protein extracts from ischemic hemispheres. ***P < 0.001, ****P < 0.0001, n = 5 per group. **C** Representative immunostaining fluorescence of MMP-9, collagen IV, and tight junction claudin-5 in the ischemic hemispheres at about 6 mm away from the frontal tip

Our previous basic and clinical studies indicate that peroxynitrite-mediated MMPs activation is a critical mechanism contributing to the BBB disruption and HT in ischemic stroke [13, 19, 39]. The activation of MMP-9 disrupts the endothelium extracellular matrix collagen IV and tight junction proteins and subsequently damaging the BBB integrity [40, 41]. Thus, we investigated the expression and activities of MMP-9 in the ischemic rat brains treated with t-PA. Gelatin zymography revealed that the t-PA-induced MMP-9 activation was blocked by AGNHW treatment (Fig. 6A, B). Immunostaining results showed that delayed t-PA treatment aggravated the reduction of extracellular matrix collagen IV whereas AGNHW treatment significantly preserved the expression of collagen IV in the microvessels of the ischemic brains (Fig. 6C). Meanwhile, t-PA treatment significantly aggravated the loss of claudin-5, a representative tight junction protein, in the microvessels of the ischemic brains whereas AGNHW treatment protected and reserved the expression of claudin-5 accordingly (Fig. 6C). Taken together, out study indicates that AGNHW could inhibit peroxynitrite-mediated MMP-9 activation and preserve extracellular matrix collagen IV and tight junction protein in ischemic brains, subsequently protecting the BBB integrity, attenuating brain edema, and HT in the transient cerebral ischemic rats with delayed t-PA treatment.

### Epiberberine, coptisine, baicalin, palmatine, berberine and baicalein are representative active compounds contributing to ONOO^−^ scavenging activity in AGNHW extract

**Fig. 7:**
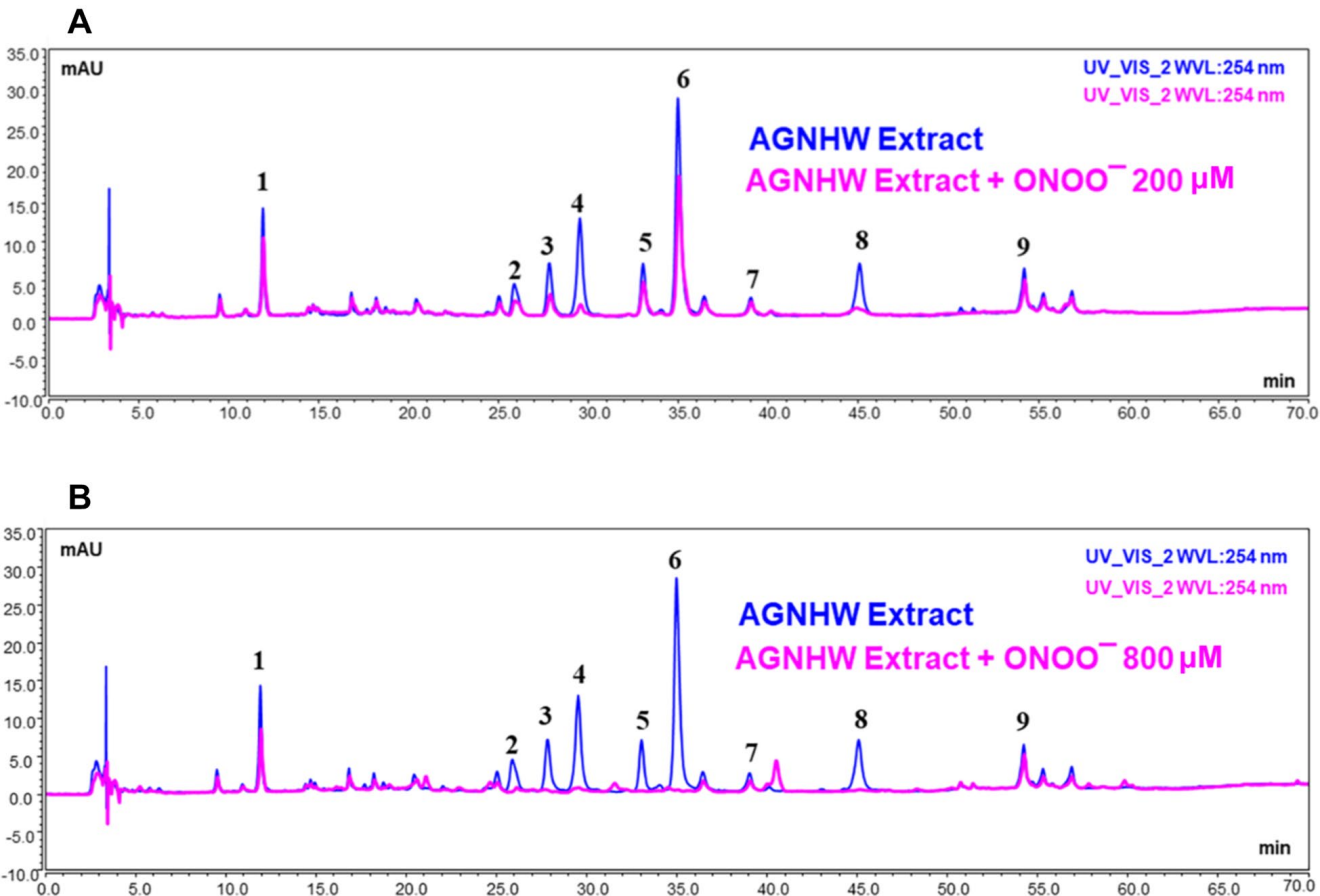
HPLC analysis of AGNHW extract reaction with peroxynitrite. **A** Representative HPLC chromatogram of AGNHW extract with or without the presence of sodium peroxynitrite at 200 µM. **B** Representative HPLC chromatogram of AGNHW extract with or without the presence of sodium peroxynitrite 800 µM. Peak 1, Geniposide; Peak 2, Epiberberine; Peak 3, Coptisine; Peak 4, Baicalin; Peak 5, Palmatine; Peak 6, Berberine; Peak 7, Wogonoisde; Peak 8, Baicalein; Peak 9, Wogonin

**Fig. 8:**
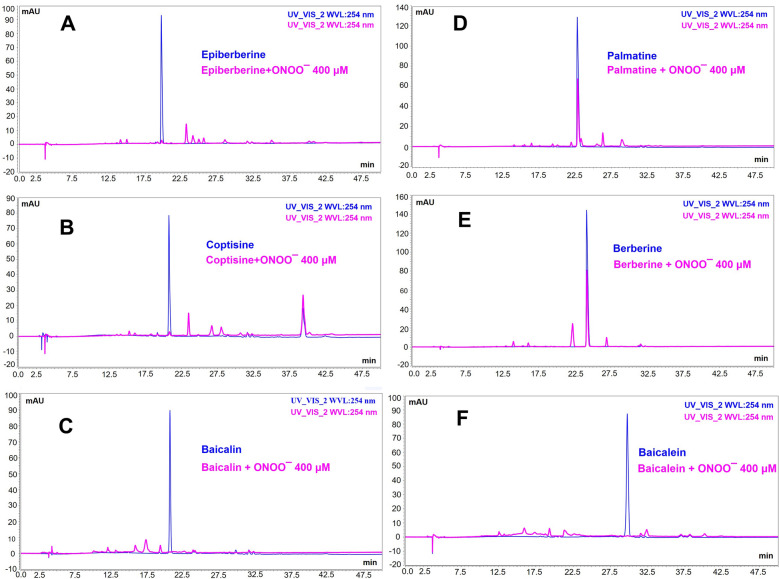
Representative HPLC chromatogram showing the reaction of each compound with peroxynitrite. Chromatograms of each compound with and without peroxynitrite (400 µM). **A** Epiberberine; **B** Coptisine; **C** Baicalin; **D** Palmatine; **E** Berberine; **F** Baicalein

Finally, we identified the active compounds with the ONOO^−^ scavenging property in the AGNHW extract. The sample was mixed with various concentrations of sodium peroxynitrite, and the HPLC chromatograms were compared to those peaks in the spectra without adding sodium peroxynitrite. The decrease of the peak area in the chromatograms indicates that the corresponding compounds were reacted with ONOO^−^. As shown in Fig. 7, six compounds (Peak 2, 3, 4, 5, 6, 8) were identified with the property of reacting with ONOO^−^ directly. These compounds included epiberberine, coptisine, baicalin, palmatine, berberine, and baicalein. Quantitative analysis revealed that adding ONOO^−^ decreased the peaks of these compounds in a dose-dependent manner, indicating that those compounds had direct reactions with ONOO^−^ (Table 2). Individually, the reactions of those compounds with ONOO^−^ were confirmed (Fig. 8). These findings suggest that epiberberine, coptisine, baicalin, palmatine, berberine and baicalein could be the main components with the ONOO^−^ scavenging activity in the AGNHW extract.

**Table 2:** Quantitative analysis on the reaction of peroxynitrite with compounds in AGNHW extract sample

ONOO^−^ (µM)	Geniposide (µg/ml)	Epiberbe-rine (µg/ml)	Coptisine (µg/ml)	Baicalin (µg/ml)	Palmatine (µg/ml)	Berberine (µg/ml)	Wogonoside (µg/ml)	Baicalein (µg/ml)	Wogonin (µg/ml)
0	21.86 ± 0.28	6.32 ± 0.11	5.64 ± 0.16	34.52 ± 0.67	6.32 ± 0.13	31.45 ± 0.41	4.31 ± 0.15	10.54 ± 0.23	4.80 ± 0.05
50	19.96 ± 0.25	5.63 ± 0.05	5.26 ± 0.11	32.57 ± 0.95	6.11 ± 0.02	28.62 ± 0.95	4.08 ± 0.09	3.95 ± 0.12	4.30 ± 0.07
100	18.04 ± 0.45	5.58 ± 0.07	5.00 ± 0.05	21.79 ± 0.78	5.89 ± 0.12	30.13 ± 0.47	3.77 ± 0.15	2.90 ± 0.14	4.32 ± 0.09
200	16.20 ± 0.69	3.86 ± 0.16	2.90 ± 0.14	4.76 ± 0.08	4.69 ± 0.20	23.20 ± 1.14	3.28 ± 0.16	2.76 ± 0.11	4.09 ± 0.18
400	14.90 ± 0.37	1.91 ± 0.09	1.09 ± 0.04	3.18 ± 0.04	2.97 ± 0.11	14.23 ± 0.41	3.01 ± 0.11	2.50 ± 0.08	3.99 ± 0.09
800	13.14 ± 0.63	n.d	n.d	n.d	n.d	n.d	2.56 ± 0.05	n.d	4.08 ± 0.11

## Discussion

In the present study, we found that AGNHW treatment at 2 h after cerebral ischemia reduced hemorrhagic transformation and improved therapeutic outcomes in the transient MCAO ischemic rats receiving delayed t-PA treatment at 5 h of cerebral ischemia. The underlying mechanisms could be attributed to scavenging peroxynitrite or/and inhibiting peroxynitrite production, preventing MMP-9 activation, and protecting tight junction proteins and microvascular integrity. Furthermore, six active compounds including epiberberine, coptisine, baicalin, palmatine, berberine and baicalein contributed to the bioactivity of AGNHW to scavenge peroxynitrite.

The disruption of BBB is a crucial pathological process in the thrombolysis-induced HT during ischemic stroke treatment [4, 5, 42, 43]. Early BBB protection could be a promising strategy for minimizing HT in ischemic stroke with t-PA treatment. In the present study, AGNHW treatment significantly preserved the expression of collagen IV and claudin-5, and reduced the BBB leakage in the ischemic brains with delayed t-PA treatment. In our previous studies, we reported that AGNHW reduced the infarct sizes in the transient cerebral ischemic rats with 2 h of MCAO ischemia plus 22 h of reperfusion [28, 29]. However, our current study revealed that AGNHW had no effects on infarct sizes in the rats with 5 h of ischemia plus 19 h of reperfusion plus delayed t-PA infusion at 5 h (Additional file 9). The inconsistent results could be due to the severe brain damages in the rat model of 5 h of MCAO cerebral ischemia with delayed t-PA treatment. As a result, AGNHW treatment could not reduce infarct sizes with such severe brain ischemia. Despite this, AGNHW showed protective effects on BBB integrity, resulting in decreased brain edema and hemorrhagic transformation, improved neurological deficit scores, and lower mortality rates.

Peroxynitrite is a crucial cytotoxic factor contributing to the BBB damage and HT in ischemic stroke with the delayed t-PA treatment [13, 19]. Pharmacologically interventions to inhibit the ONOO^−^-mediated MMPs activation protected BBB integrity, minimized HT, and improved neurological outcomes [13, 19]. We found that AGNHW inhibited ONOO^−^ production and 3-NT formation in the t-PA treated ischemic brains in vivo and the brain microvascular cells under OGD/R condition in vitro*.* AGNHW also inhibited the production of O_2_^.−^ and down-regulated the expression of NADPH oxidase subunits p47phox and p67phox, and iNOS, the enzymes responsible for O_2_^.−^ and NO production [44, 45]. Our previous study has demonstrated that peroxynitrite decomposition catalyst directly inhibited the BBB disruption and hemorrhagic transformation in the ischemia-reperfused rat brains with delayed t-PA infusion via scavenging ONOO^−^ and preventing the MMPs activation [13].

It is well known that MMPs activity contributes to both ischemic brain injury and hemorrhage transformation in ischemic stroke with t-PA treatment [46, 47]. MMPs activation disrupts extracellular matrix (ECM) to mediate ischemic brain injury [46]. Stroke patients have a significantly higher serum level of MMP-2 and MMP-9 than healthy controls [47]. Particularly, MMP-9 can disrupt tight junction proteins and extracellular matrix, causing BBB damage and HT in stroke models [40, 48–50]. Plasma MMP-9 level has been used as a biomarker for predicting BBB damage and HT in stroke patients [51, 52]. Neutrophils, microvessels and brain resident cells are major sources of MMP-9 activation contributing to hemorrhagic transformation in the presence or absence of t-PA during ischemic stroke [53, 54]. In our study, immunostaining results showed the colocalization of MMP-9 with collagen IV (Fig. 6), which suggests that endothelial cells are likely one of the major sources of MMP-9. In line with this result, our previous study found that AGNHW treatment inhibited the expression and activity of MMP-9 in ischemic brain microvessels [28]. Therefore, the inhibition of ONOO^−^-mediated MMP-9 activation could be an important underlying mechanism contributing to the effects of AGNHW on reducing the t-PA-induced BBB disruption and hemorrhagic transformation.

In addition, UPLC analysis revealed that AGNHW extract reacted with ONOO^−^ directly. We identified six active compounds to be capable of scavenging ONOO^−^, including epiberberine, coptisine, baicalin, palmatine, berberine and baicalein. It is valuable to further explore the potentials of those compounds for stroke treatment. For examples, our previous study showed that baicalin attenuated the BBB disruption and hemorrhagic transformation, and improved neurological outcome in ischemic stroke rats with delayed t-PA treatment via inhibiting ONOO^−^/MMP-9 signaling pathway [19]. Other compounds, such as baicalein, berberine and geniposide, were also reported to have neuroprotective effects in both transient and permanent MCAO stroke models [27, 55, 56]. In addition, other ingredients, like realgar and cinnabar, are also important components contributing to the neuroprotective effects against cerebral ischemia–reperfusion injury [28, 29]. Therefore, it is necessary to further study the active compounds and their synergic effects for the neuroprotection of AGNHW against ischemic brain injury.

Given that t-PA has a restrictive therapeutic window within 4.5 h after ischemic stroke onset, many patients could not reach a hospital for medical imaging diagnosis and receive treatment within such a narrow time window. Unlike other prescription drugs, AGNHW becomes a house-keeping first aid pill in Chinese Community for emergency situations. Short-term use of AGNHW at a regular dose is safe and effective for ischemic brain treatment [28, 29]. A recent meta-analysis indicates that ANGNW could improve neurologic functions with the safety for acute cerebral infarction (ACI) and acute intracerebral hemorrhage patients [57]. In this study, we demonstrate the efficacies of AGNHW to reduce the mortality rates and hemorrhagic transformation, and improve neurological functions in the transient MCAO ischemic rats with delayed t-PA treatment. Therefore, further studies with clinical trials might yield a novel therapeutic strategy to save ischemic stroke patients and extend the t-PA’s therapeutic window by using AGNHW treatment as a first aid treatment.

## Conclusion

AGNHW, a classic TCM formula, could protect the BBB integrity, reduce brain edema, prevent hemorrhagic transformation, improve neurological functions, and increase survival rates in ischemic stroke with delayed t-PA treatment. Its antioxidant property of inhibiting peroxynitrite-mediated MMP-9 activation could be one of the underlying mechanisms.

## Supplementary Information

**Additional file 1. **Chemical structures of representative standard compounds in AGNHW. These nine compounds served as controls for UPLC analysis.

**Additional file 2. **Chromatographic conditions for UPLC analysis of AGNHW samples.

**Additional file 3. **Control of the anesthesia time and body temperature during the MCAO surgery. **A,** total anesthesia time of rats during MCAO surgery in different groups. **B,** Body temperatures of rats in different groups before and after the MCAO surgery.

**Additional file 4. **Calibration curves, precision, repeatability, stability, and accuracy of the UPLC assay of nine standard compounds.

**Additional file 5. **Representative HPLC chromatograms of AGNHW extracts with different solvents.

**Additional file 6. **Quantitative analysis of the nine chemical compounds in AGNHW extract using various extraction solvents. **A,** The amount of geniposide present in various AGNHW extracts. **B**, The amount of epiberberine present in various AGNHW extracts. **C**, The amount of coptisine present in various AGNHW extracts. **D**, The amount of baicalin present in various AGNHW extracts. **E**, The amount of palmatine present in various AGNHW extracts. **F**, The amount of berberine present in various AGNHW extracts. **G**, The amount of wogonoside present in various AGNHW extracts. **H**, The amount of baicalein present in various AGNHW extracts. **I**, The amount of wogonin present in various AGNHW extracts. **J**, The total AUC areas of several AGNHW extracts' HPLC chromatograms.

**Additional file 7. **AGNHW extract had no effect on t-PA activity. The t-PA activity was measured with or without the presence of AGNHW water extract (Extract A) or AGNHW ethanol extract (Extract B), at the final concentration of 50 µg/ml. The corresponding vehicle was used as control respectively.

**Additional file 8. **Statistical analysis on relative fluorescence intensity of HEt and HKYellow AM in endothelial cells. OGD, oxygen and glucose deprivation; t-PA, tissue plasminogen activator; AGNHW, Angong Niuhuang Wan; *p < 0.05, **p < 0.01, ***p < 0.001, ****p < 0.0001. n = 4.

**Additional file 9. **Representative brain slices of TTC staining, and statistical analysis of brain infarct percentage. TTC staining revealed that t-PA or t-PA plus AGNHW treatment had no effect on the percentage of brain infarcts 24 h after stroke onset. The red color represents healthy tissue while the white color represents the brain infarct. The percentage of brain infarct was calculated as followed: [(Total area of non-ischemic side-Total area of healthy tissue in the ischemic side)/Total area of non-ischemic side]. n = 3–4.

## Data Availability

The datasets during and/or analysed during the current study available from the corresponding author on reasonable request.

## Abbreviations

3-NT: 3-Nitrotyrosine
ACI: Acute cerebral infarction
AGNHW: Angong Niuhuang Wan
BBB: Blood–brain barrier
EB: Evans blue
HT: Hemorrhagic transformation
iNOS: Inducible nitric oxide synthase
MCAO: Middle cerebral artery occlusion
M5/R19: MCAO 19 hours followed by reperfusion 19 hours
MMP-9: Matrix metalloproteinase-9
NO: Nitric oxide
O2.^−^: Superoxide
ONOO^−^: Peroxynitrite
OGD: Oxygen and glucose deprivation
RNS: Reactive nitrogen species
ROS: Reactive oxygen species
t-PA: Tissue plasminogen activator
