# Supplementary material for: Angong Niuhuang Wan reduces hemorrhagic transformation and mortality in ischemic stroke rats with delayed thrombolysis: involvement of peroxynitrite-mediated MMP-9 activation
Source: Chin Med. 2022 Apr 27;17:51. doi: 10.1186/s13020-022-00595-7 (PMC9044615; doi:10.1186/s13020-022-00595-7)
Supplement: Supplementary file 2 — Additional file 2. Chromatographic conditions for UPLC analysis of AGNHW samples. [file 13020_2022_595_MOESM2_ESM.docx]

**Additional file 2. Chromatographic conditions for UPLC analysis of AGNHW samples.**

| Mobile phase | A: Acetonitrile  B: 0.17% Phosphoric acid |  |
| --- | --- | --- |
| Gradient | Time (min)  0-2  2-15  15-23  23-45  45-50  50-60  60-70 | % A  10  10-23  23  23-35  35-45  45-55  55-90 |
| Flow rate | 1 ml/min |  |
| Detection wavelength | 240 nm |  |
| Injection volume | 10 µl |  |
| Column temperature | 30 ^0^C |  |
