# Supplementary material for: Angong Niuhuang Wan reduces hemorrhagic transformation and mortality in ischemic stroke rats with delayed thrombolysis: involvement of peroxynitrite-mediated MMP-9 activation
Source: Chin Med. 2022 Apr 27;17:51. doi: 10.1186/s13020-022-00595-7 (PMC9044615; doi:10.1186/s13020-022-00595-7)
Supplement: Supplementary file 3 — Additional file 3. Control of the anesthesia time and body temperature during the MCAO surgery. A, total anesthesia time of rats during MCAO surgery in different groups. B, Body temperatures of rats in different groups before and after the MCAO surgery. [file 13020_2022_595_MOESM3_ESM.docx]

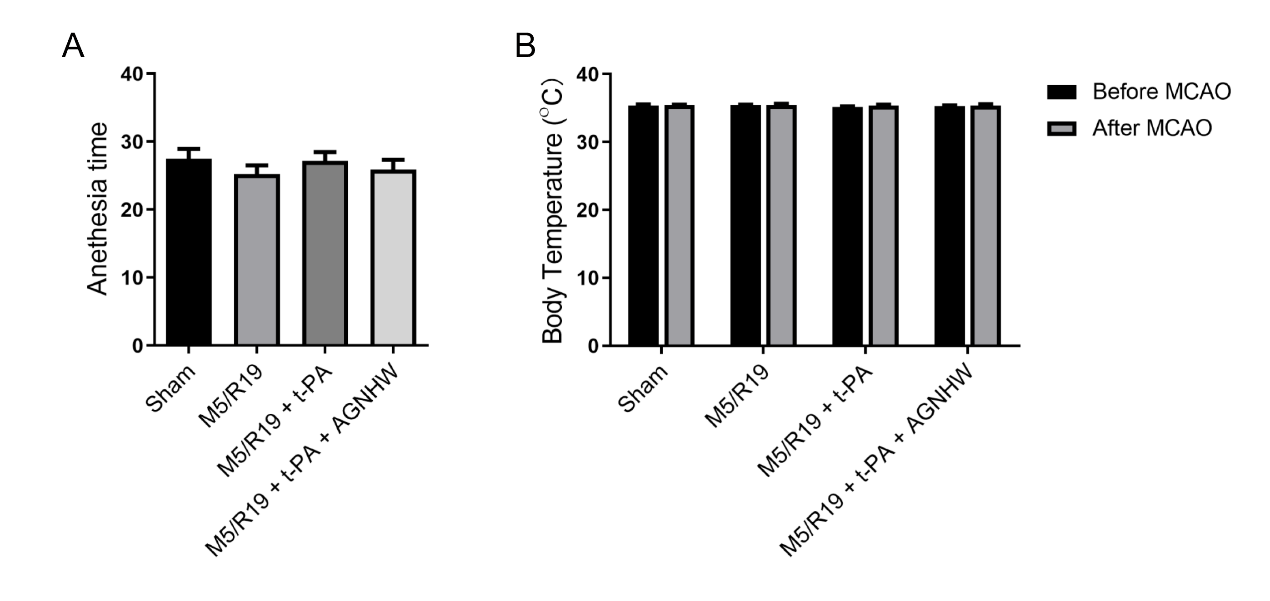


**Additional file 3. Control of the anesthesia time and body temperature during the MCAO surgery**. **A,** total anesthesia time of rats during MCAO surgery in different groups. **B,** Body temperatures of rats in different groups before and after the MCAO surgery.
