# Supplementary material for: Angong Niuhuang Wan reduces hemorrhagic transformation and mortality in ischemic stroke rats with delayed thrombolysis: involvement of peroxynitrite-mediated MMP-9 activation
Source: Chin Med. 2022 Apr 27;17:51. doi: 10.1186/s13020-022-00595-7 (PMC9044615; doi:10.1186/s13020-022-00595-7)
Supplement: Supplementary file 4 — Additional file 4. Calibration curves, precision, repeatability, stability, and accuracy of the UPLC assay of nine standard compounds. [file 13020_2022_595_MOESM4_ESM.docx]

**Additional file 4 Calibration curves, precision, repeatability, stability, and accuracy of the UPLC assay of nine standard** **compounds**

| Analyte | Linearity | | | Preci  -sion  (RSD, %, n=6) | Repeata-bility  (RSD, %,  n=6) | Stability  (RSD, %, n=6) | Spike recovery  (RSD, %, n=3) | | |
| --- | --- | --- | --- | --- | --- | --- | --- | --- | --- |
|  | Range (µg/ml) | Equation | R^2^ |  |  |  | High | Middle | Low |
| Geniposide |  | y = 0.122x-0.0024 | 0.9996 | 0.17% | 0.15% | 1.14% | 102.87 (2.30) | 100.43 (0.15) | 105.19 (1.51) |
| Epiberberine |  | y = 0.261x-0.0613 | 0.9995 | 0.16% | 0.19% | 3.69% | 99.66 (1.11) | 102.06 (1.69) | 98.14 (2.31) |
| Coptisine |  | y = 0.3985x-0.1252 | 0.9991 | 0.27% | 0.23% | 2.93% | 98.38 (0.16) | 100.74 (0.24) | 99.43 (0.95) |
| Baicalin |  | y = 0.1288x-0.0366 | 0.9996 | 0.52% | 0.53% | 4.93% | 105.19 (2.32) | 99.92 (0.66) | 99.09 (0.08) |
| Palmatine |  | y = 0.3212x-0.0654 | 0.9996 | 0.27% | 0.33% | 0.97% | 99.61 (0.84) | 102.22 (0.35) | 98.85 (1.48) |
| Berberine |  | y = 0.2973x-0.0907 | 0.9993 | 0.21% | 0.23% | 2.26% | 105.37 (0.61) | 103.30 (1.22) | 103.19 (2.43) |
| Wogonoisde |  | y = 0.204x-0.0474 | 0.9991 | 0.39% | 0.37% | 1.80% | 99.63 (0.21) | 98.98 (0.97) | 96.90 (1.50) |
| Baicalein |  | y = 0.2575x-0.1854 | 0.9990 | 0.53% | 0.95% | 1.47% | 95.03 (1.14) | 95.36 (0.39) | 99.76 (2.23) |
| Wogonin |  | y = 0.3529x-0.0985 | 0.9992 | 0.26% | 0.26% | 4.90% | 105.24 (1.55) | 100.71 (0.10) | 104.62 (0.12) |
