# Supplementary material for: Angong Niuhuang Wan reduces hemorrhagic transformation and mortality in ischemic stroke rats with delayed thrombolysis: involvement of peroxynitrite-mediated MMP-9 activation
Source: Chin Med. 2022 Apr 27;17:51. doi: 10.1186/s13020-022-00595-7 (PMC9044615; doi:10.1186/s13020-022-00595-7)
Supplement: Supplementary file 5 — Additional file 5. Representative HPLC chromatograms of AGNHW extracts with different solvents. [file 13020_2022_595_MOESM5_ESM.docx]

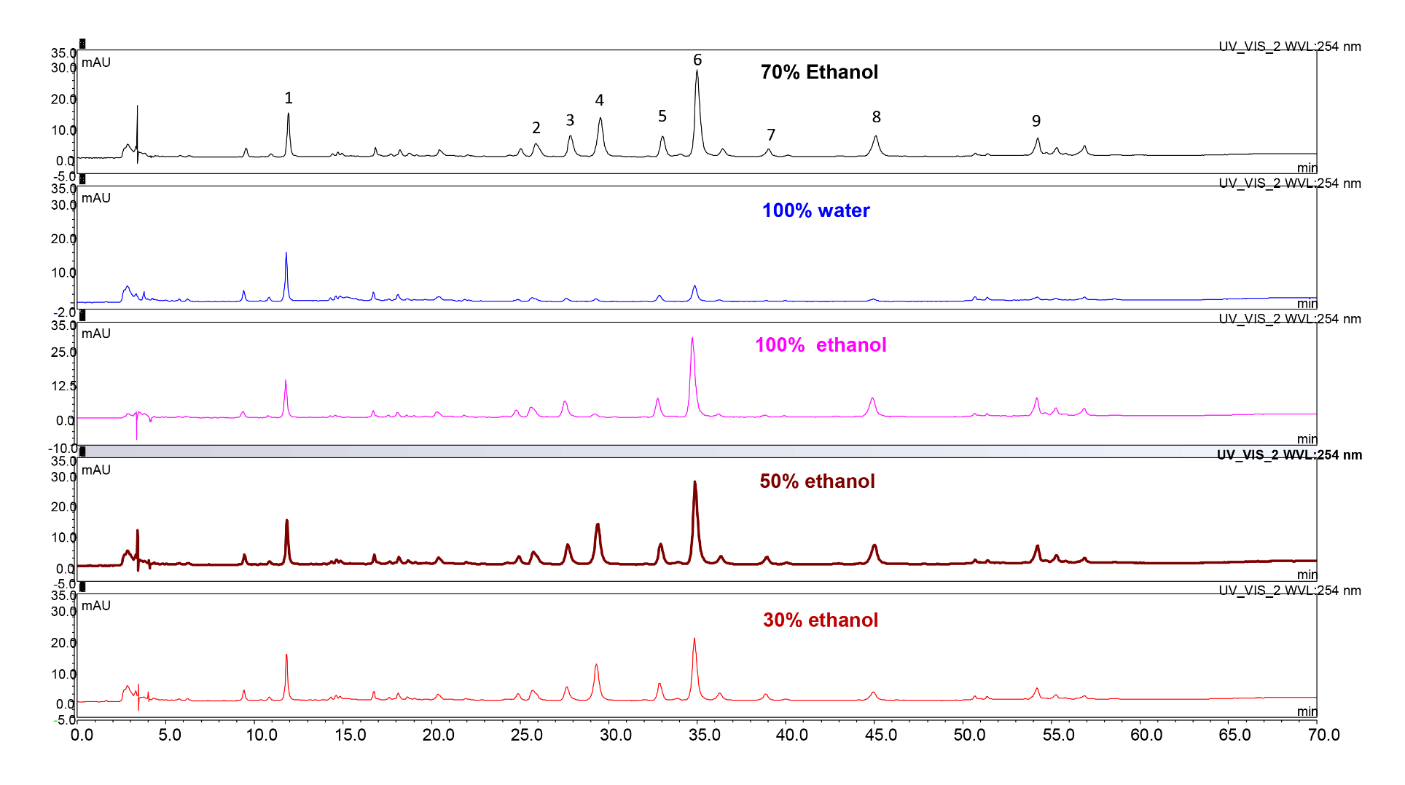


**Additional file 5. Representative HPLC chromatograms of AGNHW extracts with different solvents.**
