# Supplementary material for: Angong Niuhuang Wan reduces hemorrhagic transformation and mortality in ischemic stroke rats with delayed thrombolysis: involvement of peroxynitrite-mediated MMP-9 activation
Source: Chin Med. 2022 Apr 27;17:51. doi: 10.1186/s13020-022-00595-7 (PMC9044615; doi:10.1186/s13020-022-00595-7)
Supplement: Supplementary file 6 — Additional file 6. Quantitative analysis of the nine chemical compounds in AGNHW extract using various extraction solvents. A, The amount of geniposide present in various AGNHW extracts. B, The amount of epiberberine present in various AGNHW extracts. C, The amount of coptisine present in various AGNHW extracts. D, The amount of baicalin present in various AGNHW extracts. E, The amount of palmatine present in various AGNHW extracts. F, The amount of berberine present in various AGNHW extracts. G, The amount of wogonoside present in various AGNHW extracts. H, The amount of baicalein present in various AGNHW extracts. I, The amount of wogonin present in various AGNHW extracts. J, The total AUC areas of several AGNHW extracts' HPLC chromatograms. [file 13020_2022_595_MOESM6_ESM.docx]

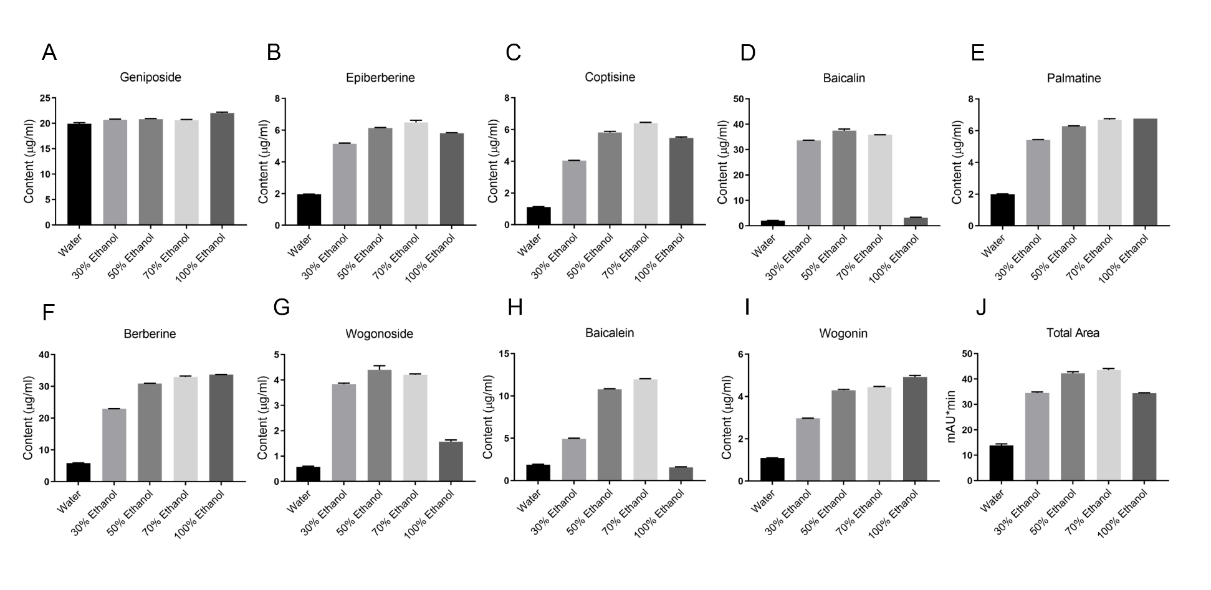


**Additional file 6. Quantitative analysis of the nine chemical compounds in AGNHW extract using various extraction solvents.** **A,** The amount of geniposide present in various AGNHW extracts. **B**, The amount of epiberberine present in various AGNHW extracts. **C**, The amount of coptisine present in various AGNHW extracts. **D**, The amount of baicalin present in various AGNHW extracts. **E**, The amount of palmatine present in various AGNHW extracts. **F**, The amount of berberine present in various AGNHW extracts. **G**, The amount of wogonoside present in various AGNHW extracts. **H**, The amount of baicalein present in various AGNHW extracts. **I**, The amount of wogonin present in various AGNHW extracts. **J**, The total AUC areas of several AGNHW extracts' HPLC chromatograms.
