# Supplementary material for: Angong Niuhuang Wan reduces hemorrhagic transformation and mortality in ischemic stroke rats with delayed thrombolysis: involvement of peroxynitrite-mediated MMP-9 activation
Source: Chin Med. 2022 Apr 27;17:51. doi: 10.1186/s13020-022-00595-7 (PMC9044615; doi:10.1186/s13020-022-00595-7)
Supplement: Supplementary file 7 — Additional file 7. AGNHW extract had no effect on t-PA activity. The t-PA activity was measured with or without the presence of AGNHW water extract (Extract A) or AGNHW ethanol extract (Extract B), at the final concentration of 50 µg/ml. The corresponding vehicle was used as control respectively. [file 13020_2022_595_MOESM7_ESM.docx]

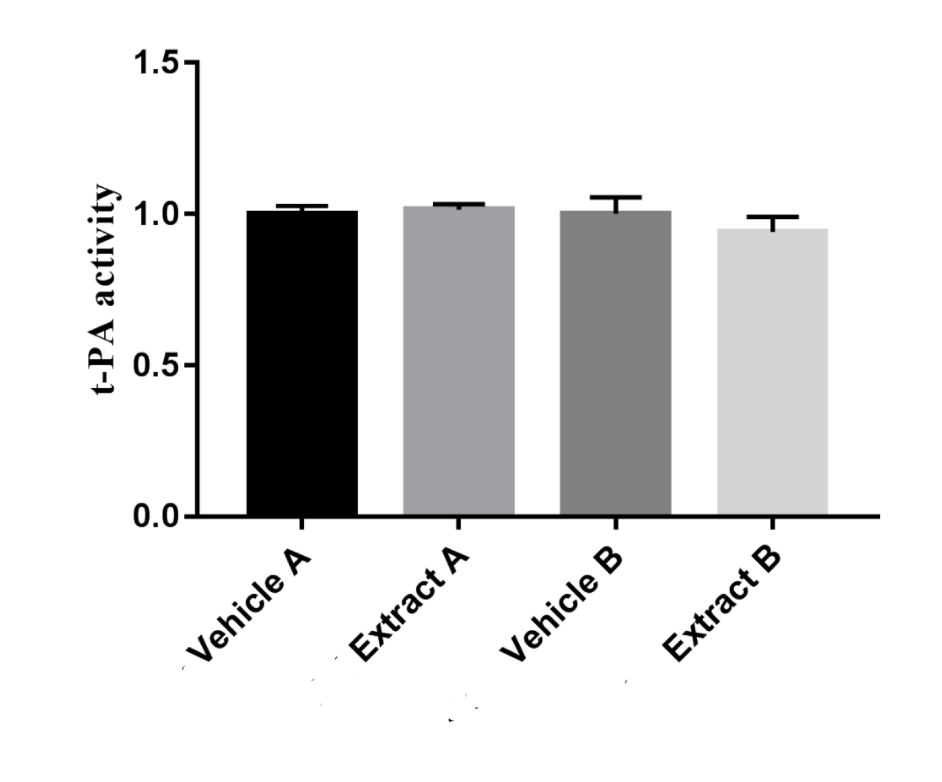


**Additional file 7. AGNHW extract had no effect on t-PA activity. The** t-PA activity was measured with or without the presence of AGNHW water extract (Extract A) or AGNHW ethanol extract (Extract B), at the final concentration of 50 µg/ml. The corresponding vehicle was used as control respectively.
