# Supplementary figures and images for: Angong Niuhuang Wan reduces hemorrhagic transformation and mortality in ischemic stroke rats with delayed thrombolysis: involvement of peroxynitrite-mediated MMP-9 activation
Source: Chin Med. 2022 Apr 27;17:51. doi: 10.1186/s13020-022-00595-7 (PMC9044615; doi:10.1186/s13020-022-00595-7)

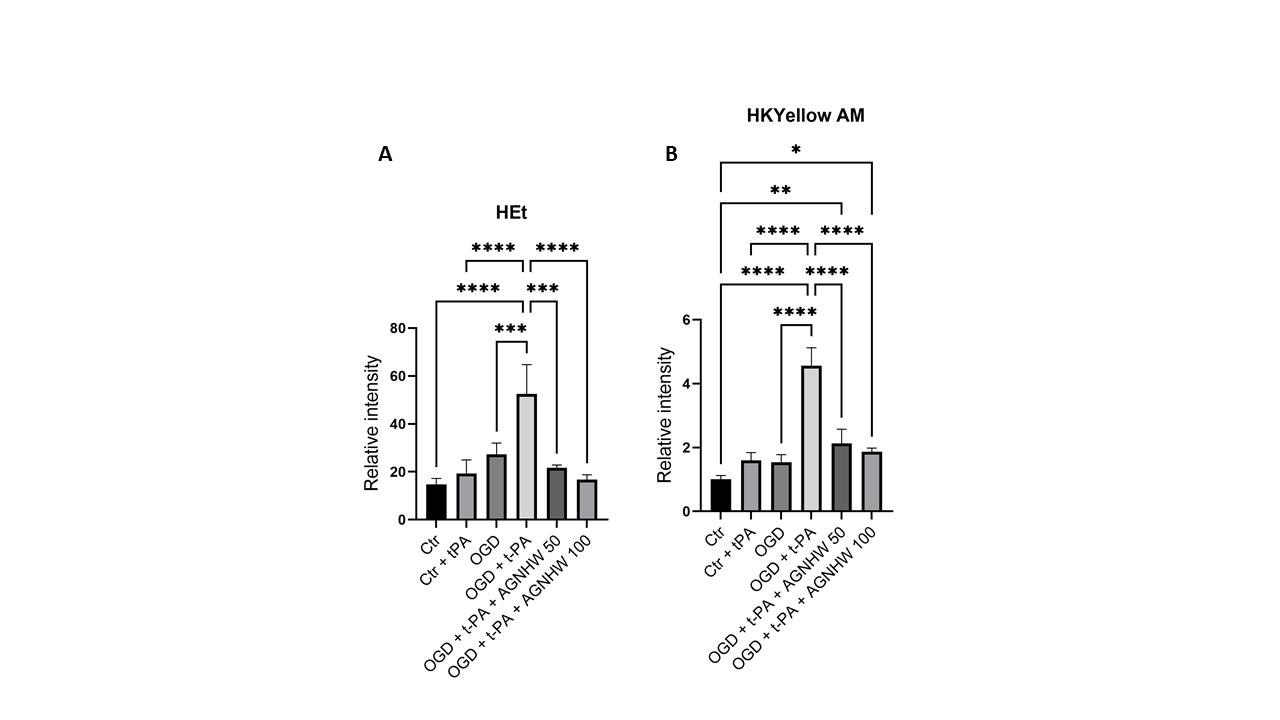

Supplement: Supplementary file 8 — Additional file 8. Statistical analysis on relative fluorescence intensity of HEt and HKYellow AM in endothelial cells. OGD, oxygen and glucose deprivation; t-PA, tissue plasminogen activator; AGNHW, Angong Niuhuang Wan; *p < 0.05, **p < 0.01, ***p < 0.001, ****p < 0.0001. n = 4. [file 13020_2022_595_MOESM8_ESM.tif]

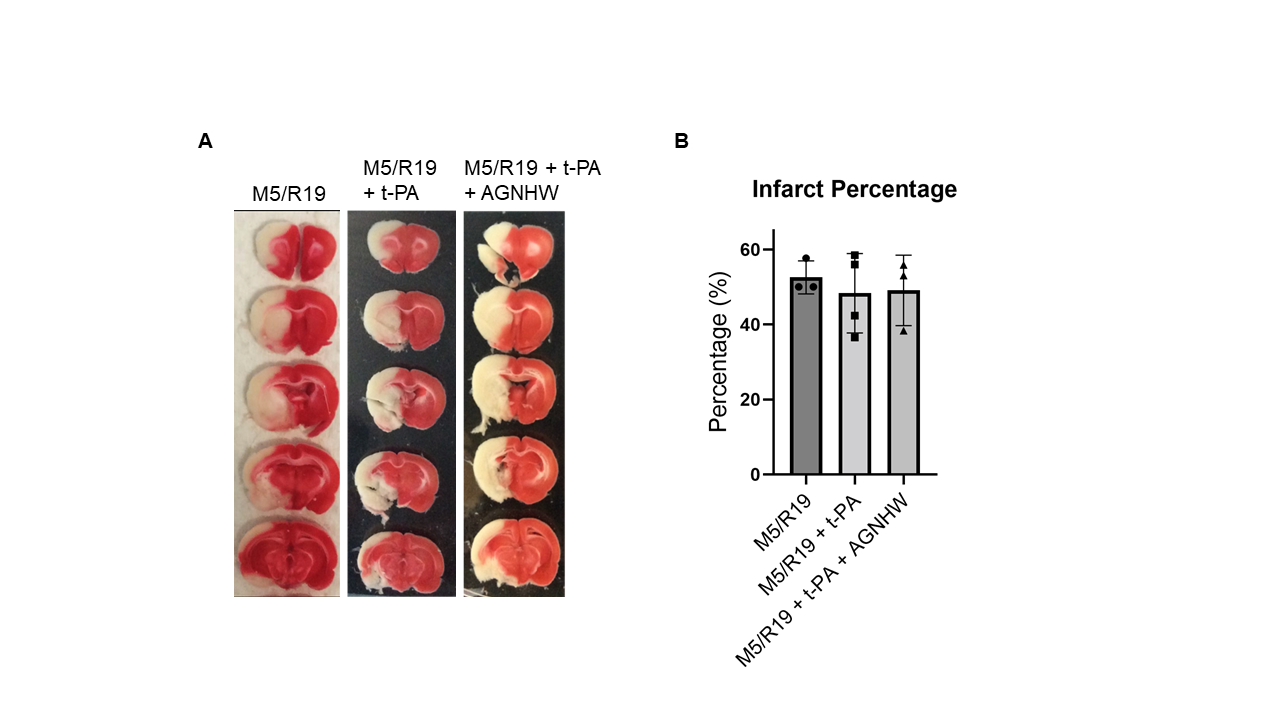

Supplement: Supplementary file 9 — Additional file 9. Representative brain slices of TTC staining, and statistical analysis of brain infarct percentage. TTC staining revealed that t-PA or t-PA plus AGNHW treatment had no effect on the percentage of brain infarcts 24 h after stroke onset. The red color represents healthy tissue while the white color represents the brain infarct. The percentage of brain infarct was calculated as followed: [(Total area of non-ischemic side-Total area of healthy tissue in the ischemic side)/Total area of non-ischemic side]. n = 3–4. [file 13020_2022_595_MOESM9_ESM.tif]
